# Prostaglandin E_2_ and F_2α_ activate the FP receptor and up-regulate cyclooxygenase-2 expression via the cyclic AMP response element

**DOI:** 10.1016/j.mce.2008.01.016

**Published:** 2008-03-26

**Authors:** Kurt J. Sales, Vivien Grant, Henry N. Jabbour

**Affiliations:** MRC Human Reproductive Sciences Unit, The Queen's Medical Research Institute, 47 Little France Crescent, Old Dalkeith Road, Edinburgh EH16 4TJ, United Kingdom

**Keywords:** Cyclooxygenase, Prostaglandin, Signal transduction, Cancer, Receptor

## Abstract

In endometrial adenocarcinomas COX-2 and F-series prostanoid (FP) receptor expression and prostanoid biosynthesis (PGE_2_ and PGF_2α_) are elevated. In the present study, we investigated the effect of PGE_2_ and PGF_2α_ on the expression of COX-2 via the FP receptor in endometrial adenocarcinoma cells stably expressing the FP receptor (FPS cells). Using chemical inhibitors of intracellular signaling pathways, reporter gene assays and quantitative RT-PCR analysis, we show that PGE_2_ and PGF_2α_ can mobilize inositol 1,4,5-trisphosphate, induce ERK1/2 phosphorylation via the phospholipase Cβ-protein kinase A-epidermal growth factor receptor pathway and induce cyclooxygenase-2 (COX-2) expression via the FP receptor. In addition we show that the PGE_2_ or PGF_2α_-regulation of COX-2 via the FP receptor is mediated via the cAMP response element (CRE) binding site on the COX-2 promoter. These data indicate that PGE_2_ and PGF_2α_ biosynthesized locally within endometrial adenocarcinomas can regulate tumor cell function in an autocrine/paracrine manner via the FP receptor.

## 1 Introduction

Prostaglandin endoperoxide (PGH) synthase or cyclooxygenase (COX) catalyses the committed step in the conversion of arachidonic acid to prostaglandins (PG) (Marnett et al., 1999). Two COX enzymes, COX-1 and COX-2, which are the targets for non-steroidal anti-inflammatory drug treatment have been characterised (Vane and Botting, 1998; Smith et al., 2000). A third COX enzyme (COX-3), a variant of COX-1 formed by retention of intron-1 and which is sensitive to acetaminophen, has been cloned more recently from canine cerebral cortex (Chandrasekharan et al., 2002).

COX-1 is constitutively expressed in many cell types and is overexpressed in certain cancers (Hwang et al., 1998; Bauer et al., 2000; Kirschenbaum et al., 2000; Sales et al., 2002). COX-2 is the more inducible form of the enzyme and is commonly associated with pathological conditions including tumorigenesis (DuBois et al., 1996; Vane et al., 1998). The existence of COX-3 protein in humans remains controversial and its role in pathologies undefined.

Following biosynthesis, prostaglandins exert their function through G protein receptor (GPCR)-mediated interaction. In the human endometrium, PGE_2_ and PGF_2α_ are the most abundantly biosynthesized prostanoids (Lumsden et al., 1983; Hofer et al., 1993). PGE_2_ exerts its autocrine/paracrine action by binding to either of four main subtypes of GPCR (EP1, EP2, EP3 and EP4) to mobilize intracellular calcium and inositol 1,4,5-trisphosphate (InsP) via G_q/11_ (EP1/EP3) or increase cAMP accumulation via G_αs_ (EP2/EP4) (Sugimoto et al., 1993; Narumiya et al., 1999). Activation of FP receptors by PGF_2α_ results in phospholipase C (PLC) activation, inositol 1,4,5-trisphosphate (InsP) hydrolysis and intracellular calcium flux (Watanabe et al., 1994). Activation of the cAMP/InsP second messenger systems by prostanoid–receptor interaction can in turn regulate target gene transcription via the phosphorylation and dephosphorylation of distal signaling pathways such as the extracellular signal-regulated kinase (ERK1/2) and phosphatidylinositol-3-kinase/protein kinase B pathways via transactivation of the epidermal growth factor receptor (EGFR) (Sheng et al., 2001; Regan, 2003; Jabbour and Sales, 2004; Sales et al., 2004a,b; Jabbour et al., 2005; Smith et al., 2006).

We have previously demonstrated elevated expression and signaling of COX-2 and FP receptor in human endometrial adenocarcinomas and have ascertained a role for PGF_2α_–FP receptor interaction in enhancing the proliferation of endometrial epithelial cells (Jabbour and Sales, 2004; Jabbour et al., 2005; Sales et al., 2004b) and promoting the expression of pro-angiogenic and inflammatory genes in endometrial adenocarcinoma cells and adenocarcinoma biopsy explants via activation of ERK1/2 (Jabbour et al., 2005; Sales et al., 2005, 2007).

Although PGE_2_ and PGF_2α_ are considered to be the endogenous ligands for EP and FP receptors, respectively, PGE_2_ can bind to the FP receptor with an affinity that is only 10–30-fold less than PGF_2α_ (Abramovitz et al., 2000). The affinity of PGF_2α_ for EP receptors is 100–300-fold less than it is for the FP receptor (Abramovitz et al., 2000).

Recently, we have shown that COX-2 and PGF_2α_ biosynthesis can be autoregulated in endometrial adenocarcinoma cells via the FP receptor (Jabbour et al., 2005). Given that both PGE_2_ and PGF_2α_ biosynthesis are elevated in endometrial pathologies (Lundstrom and Green, 1978; Lumsden et al., 1983; Sales and Jabbour, 2003) and since PGE_2_ can act as an agonist of the FP receptor, we investigated the effect of PGE_2_ and PGF_2α_ on regulation of COX-2 via the FP receptor in Ishikawa endometrial epithelial cells stably transfected with the human isoform of the FP receptor.

## 2 Materials and methods

### 2.1 Reagents

Culture medium was purchased from Life Technologies (Paisley, UK). Penicillin–streptomycin and fetal calf serum (FCS) were purchased from PAA Laboratories (Middlesex, UK). Indomethacin, phosphate buffered saline (PBS), bovine serum albumin (BSA), AL8810 (10 mM stock in ethanol), PGE_2_ and PGF_2α_ were purchased from Sigma Chemical Company (Dorset, UK). Rabbit anti-phospho-p42/44 and mouse anti-p42/44 monoclonal antibodies were purchased from Cell Signaling Technologies (New England Biolabs, Herts, UK). The Alexafluor 680 secondary antibody was purchased from Molecular Probes Inc. (Eugene, OR, USA). The IRDye™ 800 secondary antibody was purchased from Rockland Immunochemicals (Gilbertsville, PA, USA). AH6809 (10 mM Stock in dimethylsulfoxide, DMSO), U73122 (10 mM Stock in DMSO), PD98059 (18.7 mM Stock in DMSO), 4C3MQ (4-cyano-3-methylisoquinoline; 10 mM stock in DMSO), GF109203x (10 mM stock in DMSO) and AG1478 (10 mM stock in DMSO) were purchased from Calbiochem (Nottingham, UK). EP4 antagonist (ONOAE2227, 10 mM stock in ethanol, used at a final concentration of 1 μM) was chemically synthesised by Charnwood Molecular Ltd. (Leics, UK).

Doses of chemical inhibitors described in Table 1 and antibodies were determined empirically by titration using the manufacturer's guidelines.

### 2.2 Cell culture

Ishikawa endometrial adenocarcinoma cells were obtained from the European Collection of Cell Culture (Wiltshire, UK). Stable FP transfectant cells were constructed, characterised and maintained as described (Sales et al., 2005), with the addition of a maintenance dose of 200 μg/ml G418.

### 2.3 Total inositol phosphate (IP) assays

Total inositol phosphate (InsP) production was measured in Ishikawa WT and FPS cells and assayed as described previously (Berg et al., 1994; Sales et al., 2005). Cells were treated either with vehicle, PGE_2_ or PGF_2α_ in the absence or presence of receptor antagonist or chemical inhibitor as shown in the figure legends. Data are presented as mean ± S.E.M. from at least 3 independent experiments.

### 2.4 cAMP assay

cAMP accumulation was determined in response to administration of vehicle, PGE_2_ or PGF_2α_ in the absence or presence of receptor antagonist or chemical inhibitor as shown in the figure legend and was performed as described previously (Sales et al., 2002). Briefly, cells (2 × 10^5^) were seeded and allowed to attach overnight. The following day, the cells were serum starved by incubating with fresh serum-free medium containing 8.4 μM indomethacin for at least 16 h. Thereafter the culture medium was removed and replaced with serum-free medium containing 3-isobutyl-1-methyl xanthine (IBMX; Sigma) to a final concentration of 1 mM and receptor antagonist/chemical inhibitor for 30 min at 37 °C. Cells were then stimulated with ligand for the time indicated in the figure legend. Following stimulation, cells were lysed in 0.1 M HCl. cAMP concentration was quantified by ELISA using a cAMP kit (Biomol, Affiniti, Exeter, UK) according to the manufacturer's protocol and normalised to protein concentration of the lysate. Protein concentrations were determined using protein assay kits (Bio-Rad Laboratories, Hemel Hamstead, UK). Data are presented as mean ± S.E.M. from at least 4 independent experiments.

### 2.5 In-cell Western detection

Cell signaling to ERK1/2 was investigated using an In-cell Western assay. Approximately, 20,000 cells were seeded per well in a 96-well microtitre plate and allowed to adhere overnight at 37 °C. The following day cells were starved by serum withdrawal in serum-free culture medium containing 8.4 μM indomethacin for at least 16 h at 37 °C. Cells were incubated for 30 min with vehicle, receptor antagonist or chemical inhibitor as described in the figure legend. Thereafter cells were stimulated with either vehicle PGE_2_ or PGF_2α_ in the absence or presence of receptor antagonist or chemical inhibitor for the time indicated in the figure legend. Following stimulation, cells were washed with ice-cold PBS, fixed in 3.7% (v/v) Formaldehyde for 20 min at room temperature, and permeabilised with 0.1% Triton X-100 in PBS. Cells were then blocked for 45 min at room temperature with Odyssey Blocking buffer™ (LI-COR Biosciences, Cambridge, UK) before overnight incubation with primary rabbit phospho-p42/44 and goat p42/44 antibodies (diluted 1:100 in Odyssey blocking buffer) at 4 °C. The following day, cells were washed and incubated with the goat anti-rabbit Alexafluor 680 (1:200) and goat anti-mouse IRDye™ 800 (1:800) for 60 min at room temperature.

Immunoreactive proteins were detected and quantified using the Odyssey infrared imaging system (LI-COR Biosciences). ERK1/2 phosphorylation was calculated by dividing the value obtained from the phosphorylated ERK1/2 channel (700 nm) by the value obtained from total ERK1/2 channel (800 nm) and expressed as fold above vehicle controls. Results are expressed as mean ± S.E.M. from at least 3 independent experiments performed in triplicate.

### 2.6 Taqman quantitative RT-PCR

COX-2 mRNA expression in FPS cells was measured by quantitative RT-PCR analysis. FPS cells were starved by serum withdrawal for at least 12 h in serum-free medium containing 8.4 μM indomethacin. Thereafter medium was removed and replaced with fresh medium containing indomethacin with either vehicle, PGE_2_ or PGF_2α_ in the absence or presence of receptor antagonist or chemical inhibitor as described in the figure legend. RNA was extracted using Tri-reagent (Sigma) following the manufacturers guidelines. Once extracted and quantified, RNA samples were reverse transcribed and subjected to RT-PCR analysis using an ABI Prism 7900 (Jabbour et al., 2005). COX-2 primers and probe for quantitative PCR were designed using the PRIMER express program (PE Applied Biosystems, Warrington, UK) as described previously (Jabbour et al., 2005). Data were analysed and processed using Sequence Detector v1.6.3 (PE Applied Biosystems). Expression of COX-2 was normalised to RNA loading for each sample using the 18S ribosomal RNA as an internal standard. Results are expressed as fold increase above vehicle treated from at least 4 independent experiments and represented as mean ± S.E.M.

### 2.7 Transfection of CRE and COX-2 promoter with deletions and mutations

Ishikawa FPS cells were transiently transfected for 6 h using a liposomal transfection system (Superfect, Qiagen, Crawley, UK). Transfections were performed with CRE-Luciferase a specific *cis*-acting DNA binding sequence of the cAMP response element ligated with a Luciferase reporter plasmid (Clontech Laboratories, BD Bioscience, Cowley, UK) or the COX-2 promoter, performed using C2.1 (−917 to +49) 966 base pair (bp) fragment of the COX-2 promoter and C2.1 with a series of deletions or site specific mutations ligated with a Luciferase reporter plasmid pGL3 basic (Promega, Southampton, UK) as described in Bradbury et al. (2003); kindly supplied by Dr. Robert Newton, BioMedical Research Institute, Department of Biological Sciences, The University of Warwick, UK. Deletions consisted of Dra (−625/+49) 674 bp, Sty (−358/+49) 407 bp, Alu (190/+49) 239 bp and RSA (−86/+49) 135 bp fragments. The description of each clone is based on the restriction site used to generate the construct (Dra, Alu, Sty, RSA, HIN). Mutations consisted of CRE (−59/−53), a mutation in the cAMP response element. The HIN (−79/+53) 200 bp and HIN CRE-mutation (HINcrem; −79/+53, 200 bp) fragments were generated by polymerase chain reaction (PCR) using the Sty and Stycrem cDNA (Bradbury et al., 2003) as a template.

Amplification of HIN and HINcrem was carried out using standard PCR mix containing forward 5′-AAGGCGGAAAGAAACAGTCA-3′ and reverse 5′-AACAGTACCGGAATGCC-AAG-3′ primers containing the HindIII restriction site at the start for ease of cloning. To amplify by PCR, sample mix was denatured at 94 °C for 5 min and subjected to 40 cycles of 94 °C for 1 min, 58 °C for 1 min and 68 °C for 1 min, with a final extension step of 68 °C for 7 min. After amplification, samples were cooled to 4 °C and visualised on 1% agarose gels. The PCR product was ligated into the pCR^®^II-TOPO vector (Invitrogen, De Schelp, The Netherlands) followed by sequencing in both directions using a PE Applied Biosystems 373A automated sequencer. The HIN and HINcrem cDNA was ligated into the pGL3 basic (Promega) expression vector followed by sequencing.

### 2.8 Luciferase reporter assays

CRE or COX-2 promoter firefly Luciferase reporter vectors were co-transfected into Ishikawa FPS cells in triplicate with an internal control pRL-TK (containing the Renilla Luciferase coding sequence; Promega) as described (Jabbour et al., 2005). The following day the cells were serum-starved for at leased 16 h 37 °C with 8.4 μM indomethacin prior to stimulation for 4 h with vehicle, PGE_2_ or PGF_2α_ in the absence or presence of receptor antagonist or chemical inhibitor as described in the figure legend. The activity of both firefly and Renilla Luciferase was determined using the dual Luciferase assay kit (Promega) and total Luciferase activity was determined by dividing the relative light units generated by the firefly Luciferase by the relative light units generated by the Renilla Luciferase in the same reaction. Fold increase in Luciferase activity was calculated by dividing the total Luciferase activity in cells treated with PGE_2_ or PGF_2α_ in the absence or presence of receptor antagonist or chemical inhibitor by the total Luciferase activity in cells treated with vehicle. Data are presented as mean ± S.E.M. from at least 4 independent experiments.

### 2.9 Statistics

Data were subjected to statistical analysis with ANOVA and Fishers protected least significant difference tests (Statview 5.0; Abacus Concepts Inc., USA).

## 3 Results

### 3.1 PGE_2_ and PGF_2α_ mobilize inositol 1,4,5-trisphosphate in FPS cells

We have previously reported elevated FP receptor expression in endometrial adenocarcinomas (Sales et al., 2004b) and constructed and characterized an endometrial adenocarcinoma (Ishikawa) cell line expressing FP receptor (FPS cells) to the levels observed in endometrial adenocarcinomas (Sales et al., 2005). In this latter study we showed that PGF_2α_-FP receptor interaction in FPS cells increases the hydrolysis of InsP in FPS cells via G_q/11_ to a greater extent than in wild-type (WT) Ishikawa cells (Sales et al., 2005).

**Fig. 1:**
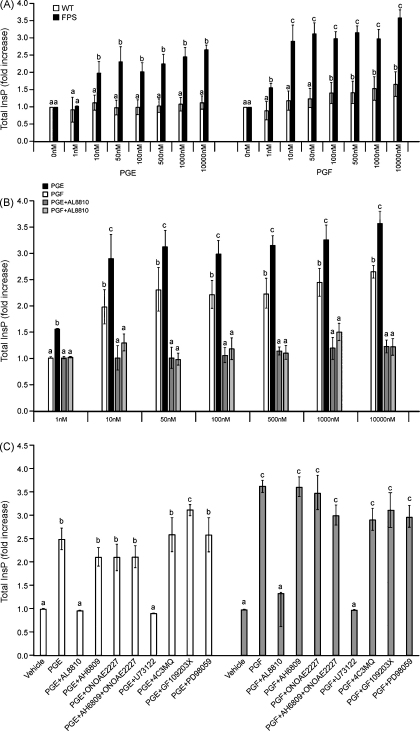
(A) Total inositol phosphate (InsP) production was assessed in Ishikawa WT or FPS cells treated with increasing doses of PGE_2_ or PGF_2α_ for 1 h at 37 °C. (B) Total InsP production in FPS cells treated with increasing doses of PGE_2_ or PGF_2α_ in the absence or presence of 50 μM of the specific FP receptor antagonist AL8810 for 1 h at 37 °C. (C) Total InsP production in FPS cells treated with 100 nM PGE_2_ or 100 nM PGF_2α_ in the absence/presence of the FP receptor antagonist AL8810 (50 μM), EP2 receptor antagonist (AH6809; 10 μM), EP4 receptor antagonist (ONOAE2227; 1 μM) or chemical inhibitors of phospholipase Cβ (U73122, 10 μM), protein kinase A (4C3MQ, 1 μM), protein kinase C (GF109203x, 10 μM) or ERK1/2 kinase (PD98059, 50 μM) for 1 h at 37 °C. Data are presented as mean ± S.E.M. b is significantly different from a, c is significantly different from a and b; *P* < 0.05.

In the present study, we found that PGE_2_ could also dose-dependently mobilize InsP in FPS cells (Fig. 1A; *P* < 0.05). No such increase in InsP production was observed in WT cells in response to PGE_2_, but a modest increase was observed in response to PGF_2α_ (Fig. 1A). Co-incubation of FPS cells with the specific FP receptor antagonist AL8810 abolished the PGE_2_ or PGF_2α_-mediated increase in InsP production at all concentrations of ligand administered (Fig. 1B, *P* < 0.05). These results suggest that the PGE_2_-mediated InsP hydrolysis in FPS cells was via the FP receptor.

We further confirmed that the InsP hydrolysis in response to 100 nM PGE_2_ or 100 nM PGF_2α_ was not mediated via activation of either of the endogenous EP2 or EP4 receptors or intracellular signaling cascades downstream of phospholipase Cβ (PLC) as neither the specific EP2 receptor antagonist (AH6809), the EP4 receptor antagonist (ONOAE2227; Fig. 1C, *P* < 0.05) nor the chemical inhibitors of the protein kinase A (PKA; 4-cyano-3-methylisoquinoline; 4C3MQ), protein kinase C (PKC; GF109203x) and ERK1/2 (PD98059) signaling pathways (Fig. 1C, *P* < 0.05, *P* < 0.05) inhibited the PGE_2_ or PGF_2α_-mediated InsP production. As shown for the specific FP receptor antagonist (AL8810), the PLC inhibitor (U73122) also abolished the InsP produced in response to treatment of FPS cells with 100nM PGE_2_ or 100nM PGF_2α_ (Fig. 1C, *P* < 0.05) further demonstrating the InsP production in response to ligand activation was mediated via the FP receptor-PLC pathway.

### 3.2 PGE_2_ and PGF_2α_ promote cyclic adenosine-3,5-monophosphate (cAMP) in FPS cells

**Fig. 2:**
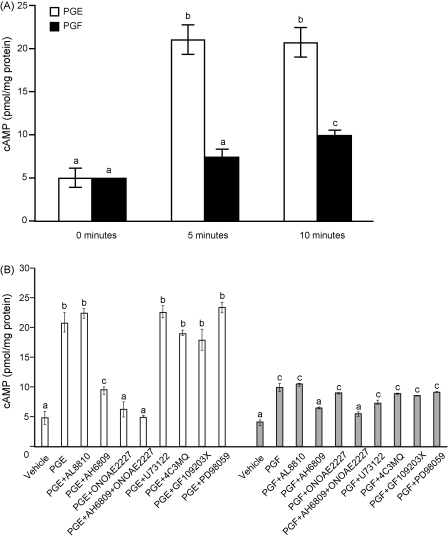
(A) cAMP accumulation in FPS cells in response to treatment with 100 nM PGE_2_ or 100 nM PGF_2α_ for 0, 5 and 10 min. (B) cAMP accumulation in FPS cells in response to treatment with 100 nM PGE_2_ or 100 nM PGF_2α_ for 10 min in the presence/absence of the FP receptor antagonist AL8810 (50 μM), EP2 receptor antagonist (AH6809; 10 μM), EP4 receptor antagonist (ONOAE2227; 1 μM) or chemical inhibitors of phospholipase Cβ (U73122, 10 μM), protein kinase A (4C3MQ, 1 μM), protein kinase C (GF109203x, 10 μM) or ERK1/2 kinase (PD98059, 50 μM). Data are presented as mean ± S.E.M. b is significantly different from a and c is significantly different from a and b; *P* < 0.05.

In addition to elevated FP receptor, Ishikawa FPS cells also express basal levels of EP2 and EP4 receptor, but not detectable EP1 receptor (data not shown), which couple to G_s_ and mobilize intracellular cAMP. We investigated cAMP accumulation in FPS cells in response to treatment with 100 nM PGE_2_ or 100 nM PGF_2α_ for 0, 5 or 10 min (Fig. 2A). We found that 100 nM PGE_2_ rapidly mobilized intracellular cAMP at 5 and 10 min (Fig. 2A; *P* < 0.05). By contrast 100 nM PGF_2α_ modestly increased cAMP following 10 minutes of treatment only (Fig. 2A; *P* < 0.05). We confirmed that the cAMP produced in response to 100 nM PGE_2_ or 100 nM PGF_2α_ was not mediated by an intracellular mechanism via activation of the FP receptor or PLC, PKA, PKC or ERK1/2 signaling cascades as neither the specific FP receptor antagonist (AL8810; Fig. 2B; *P* < 0.05) nor the chemical inhibitors of the PKA (4C3MQ), PKC (GF109203x) or ERK1/2 (PD98059) signaling pathways (Fig. 2B; *P* < 0.05) inhibited the PGE_2_ or PGF_2α_-mediated cAMP production following ligand stimulation for 10 min. However, co-treatment of FPS cells with the EP2 receptor antagonist (AH6809) or EP4 receptor antagonist (ONOAE2227) significantly reduced the PGE_2_- or PGF_2α_-mediated cAMP response. Moreover, the EP2 (AH6809) and EP4 (ONOAE2227) receptor antagonist in combination totally abolished the PGE_2_, or PGF_2α_-mediated cAMP response following 10 min of ligand stimulation (Fig. 2B; *P* < 0.05).

### 3.3 PGE_2_ and PGF_2α_ signaling cascades converge on ERK1/2

**Fig. 3:**
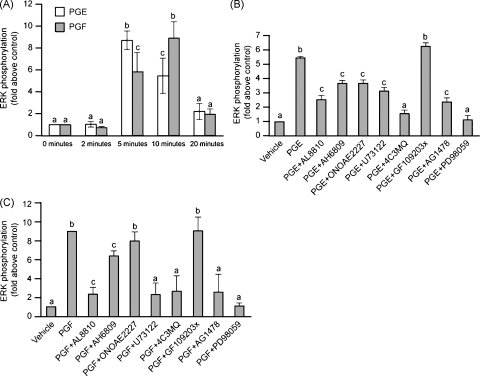
(A) ERK1/2 phosphorylation in FPS cells in response to treatment with 100 nM PGE_2_ or 100 nM PGF_2α_ for 0, 2, 5, 10 and 20 min. ERK1/2 phosphorylation in FPS cells in response to treatment with 100 nM PGE_2_ (B) or 100 nM PGF_2α_ (C) for 10 min in the presence/absence of the FP receptor antagonist AL8810 (50 μM), EP2 receptor antagonist (AH6809; 10 μM), EP4 receptor antagonist (ONOAE2227; 1 μM) or chemical inhibitors of phospholipase Cβ (U73122, 10 μM), protein kinase A (4C3MQ, 1 μM), protein kinase C (GF109203x, 10 μM), EGFR kinase (AG1478, 200 nM) or ERK1/2 kinase (PD98059, 50 μM). Data are presented as mean ± S.E.M. b is significantly different from a and c is significantly different from a and b; *P* < 0.05.

The effect of PGE_2_ or PGF_2α_ on the activation of the downstream extracellular signal-regulated kinase (ERK1/2) signaling pathway was determined after treatment of FPS cells with vehicle, 100 nM PGE_2_ or 100nM PGF_2α_ for 0, 5, 10 and 20 min (Fig. 3A; *P* < 0.05). Stimulation of FPS cells with PGE_2_ or PGF_2α_ caused a rapid time-dependent activation of ERK1/2 (Fig. 3A; *P* < 0.05). The peak of ERK1/2 activation was observed after 5 min in FPS cells treated with 100 nM PGE_2_ and 10 min in cells treated with 100 nM PGF_2α_ (Fig. 3A, *P* < 0.05).

We next evaluated the effect of the FP receptor antagonist (AL8810), EP2 receptor antagonist (AH6809), EP4 receptor antagonist (ONOAE2227) and chemical inhibitors of PLC (U73122), PKC (GF109203x), PKA (4C3MQ), EGFR (AG1478) and ERK1/2 kinase (MEK; PD98059) on the PGE_2_ or PGF_2α_-induced activation of ERK1/2 signaling. As observed in Fig. 3A, ERK1/2 phosphorylation was significantly elevated in FPS cells treated for 10 min with PGE_2_ (Fig. 3B, *P* < 0.05). The PGE_2_-induced elevation in ERK1/2 activation was significantly inhibited by co-treatment of FPS cells with FP receptor antagonist (AL8810), EP2 receptor antagonist (AH6809), EP4 receptor antagonist (ONOAE2227) and PLC inhibitor (U73122) and abolished by treatment of cells with the PKA (4C3MQ), EGFR kinase (AG1478) and ERK1/2 kinase (PD98059) inhibitors, but not the PKC inhibitor (GF109203x; Fig. 3B, *P* < 0.05). The PGF_2α_-induced elevation in ERK1/2 activation was significantly inhibited by co-treatment of cells with the EP2 antagonist (AH6809), but not the EP4 receptor antagonist (ONOAE2227) or PKC inhibitor (GF109203x), and was abolished by treatment with the FP receptor antagonist (AL8810), and inhibitors of PLC (U73122), PKA (4C3MQ), EGFR kinase (AG1478) and ERK1/2 kinase (PD98059; Fig. 3B, *P* < 0.05).

### 3.4 Activation of COX-2 Luciferase reporter and mRNA by PGE_2_ and PGF_2α_

**Fig. 4:**
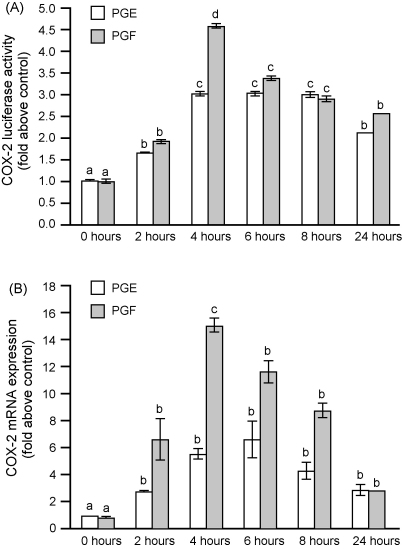
COX-2 Luciferase activity (A) and mRNA expression (B) in FPS cells in response to treatment with 100 nM PGE_2_ or 100 nM PGF_2α_ for 0, 2, 4, 6, 8 and 24 h. Data are presented as mean ± S.E.M. b is significantly different from a, c is significantly different from a and b; *P* < 0.05.

The role of PGE_2_ or PGF_2α_ on the activation of COX-2 in FPS cells was investigated by Luciferase reporter gene assay (Fig. 4A) and quantitative RT-PCR analysis (Fig. 4B). Treatment of FPS cells with PGE_2_ or PGF_2α_ caused a significant time-dependent increase in COX-2 Luciferase reporter activity (Fig, 4A; *P* < 0.05) and mRNA expression (Fig. 4B; *P* < 0.05) which peaked at 4–6 h. The PGF_2α_-induced increase in COX-2 reporter gene activation (Fig. 4A) and mRNA expression (Fig. 4B) was greater than that induced by PGE_2_ (*P* < 0.05).

### 3.5 COX-2 expression is mediated via activation of the ERK pathway

**Fig. 5:**
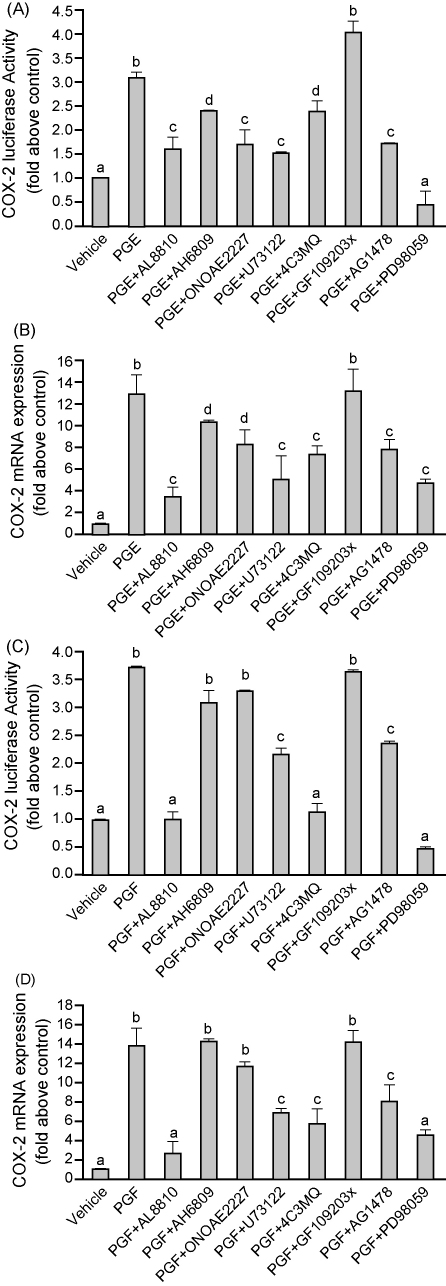
COX-2 Luciferase activity (A, C) and mRNA expression (B, D) in FPS cells in response to treatment with 100 nM PGE_2_ (A and B) or 100 nM PGF_2α_ (C and D) for 4 h in the presence/absence of the FP receptor antagonist AL8810 (50 μM), EP2 receptor antagonist (AH6809; 10 μM), EP4 receptor antagonist (ONOAE2227; 1 μM) or chemical inhibitors of phospholipase Cβ (U73122, 10 μM), protein kinase A (4C3MQ, 1 μM), protein kinase C (GF109203x, 10 μM), EGFR kinase (AG1478, 200 nM) or ERK1/2 kinase (PD98059, 50 μM). Data are presented as mean ± S.E.M. b is significantly different from a and c is significantly different from a and b, d is significantly different from a, b and c; *P* < 0.05.

We set out to determine the signaling pathways mediating COX-2 expression in FPS cells. Cells were treated with vehicle, 100 nM PGE_2_ or 100 nM PGF_2α_ in the presence/absence of the FP receptor antagonist (AL8810), EP2 receptor antagonist (AH6809), EP4 receptor antagonist (ONOAE2227), or chemical inhibitors of PLC (U73122), PKA (4C3MQ), PKC (GF109203x), EGFR kinase (AG1478) or ERK1/2 kinase (PD98059). COX-2 promoter activation and mRNA expression was determined using a reporter cDNA construct containing the full length COX-2 promoter cDNA fused upstream of the firefly Luciferase reporter (C2.1; Fig. 5A and C) and real-time quantitative RT-PCR analysis (Fig. 5B and D), respectively. The PGE_2_-induced elevation in COX-2 Luciferase (Fig. 5A) and mRNA expression (Fig. 5B) was significantly reduced by treatment of FPS cells with the FP receptor antagonist (AL8810), EP2 receptor antagonist (AH6809), EP4 receptor antagonist (ONOAE2227), and chemical inhibitors of PLC (U73122), PKA (4C3MQ), EGFR kinase (AG1478) and ERK1/2 kinase (PD98059), but not the PKC inhibitor (GF109203x; *P* < 0.05). The PGF_2α_-induced elevation in COX-2 Luciferase (Fig. 5C) and mRNA expression (Fig. 5D) was significantly inhibited by co-treatment of cells with the FP receptor antagonist (AL8810), and chemical inhibitors of PLC (U73122), PKA (4C3MQ), EGFR kinase (AG1478) and ERK1/2 kinase (PD98059), but not the EP2 receptor antagonist (AH6809), EP4 receptor antagonist (ONOAE2227) or PKC inhibitor (GF109203x; Fig. 5C and D, *P* < 0.05).

### 3.6 Mutation of the CRE-binding site in the COX-2 promoter inhibits PGE_2_ and PGF_2α_-mediated Luciferase activity

**Fig. 6:**
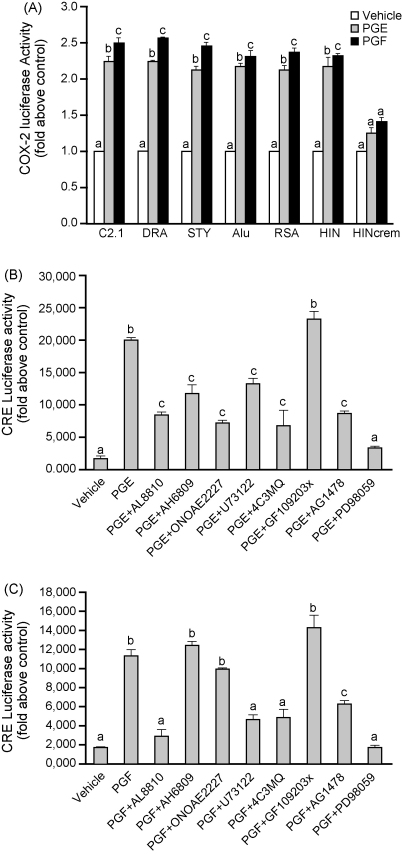
(A) COX-2 Luciferase activity in FPS cells transiently transfected with the full length COX-2 promoter C2.1 or series of 5’deletions (DRA, STY, ALU, RSA, HIN) and HIN with a mutation in the cAMP response element (HINcrem; −59/−53). FPS cells were treated with vehicle, 100 nM PGE_2_ or 100nM PGF_2α_ for 4 h. CRE Luciferase activity in FPS cells transiently transfected with the *cis*-acting DNA binding sequence of the cAMP response element (CRE). FPS cells were treated with 100 nM PGE_2_ (B) or 100 nM PGF_2α_ (C) for 4 h in the presence/absence of the FP receptor antagonist AL8810 (50 μM), EP2 receptor antagonist (AH6809; 10 μM), EP4 receptor antagonist (ONOAE2227; 1 μM) or chemical inhibitors of phospholipase Cβ (U73122, 10 μM), protein kinase A (4C3MQ, 1 μM), protein kinase C (GF109203x, 10 μM), EGFR kinase (AG1478, 200 nM) or ERK1/2 kinase (PD98059, 50 μM). Data are presented as mean ± S.E.M. b is significantly different from a and c is significantly different from a and b; *P* < 0.05.

To determine which transcription factors were involved in mediating COX-2 expression in response to PGE_2_ or PGF_2α_, FPS cells were transiently transfected with the 966 bp full length COX-2 promoter (C2.1) or COX-2 promoter containing a series of deletions as described in Bradbury et al. (2003). PGE_2_ or PGF_2α_ increased COX-2 Luciferase activity by 2.2 ± 0.1 and 2.6 ± 0.1-fold, respectively (Fig. 6A; *P* < 0.05). There was no significant reduction in Luciferase activity with any of the sequential 5′ deletions compared with the C2.1, suggesting that even the smallest HIN fragment of the COX-2 promoter, which contains only the CRE, was able to induce COX-2 Luciferase activity (Fig. 6A). Transfection of FPS cells with the HIN COX-2 promoter fragment with a mutated CRE (HINcrem; −59/−53) abolished the COX-2 activity induced by PGE_2_ or PGF_2α_ (Fig. 6A; *P* < 0.05), suggesting that this factor is necessary for COX-2 induction by prostanoids.

### 3.7 CRE is activated by PGE_2_ and PGF_2α_ signaling to ERK

We further confirmed the signaling pathways activating the CRE in FPS cells transfected with cDNA construct containing a specific *cis*-acting DNA binding sequence of the cAMP response element ligated with a Luciferase reporter plasmid. Cells were treated with vehicle, 100 nM PGE_2_ or 100 nM PGF_2α_ in the presence/absence of the FP receptor antagonist (AL8810), EP2 receptor antagonist (AH6809), EP4 receptor antagonist (ONOAE2227), PLC inhibitor (U73122), PKA inhibitor (4C3MQ), PKC inhibitor (GF109203x), EGFR kinase inhibitor (AG1478) or ERK1/2 inhibitor (PD98059) for 4 h. CRE Luciferase activity in FPS cells was significantly elevated in response to PGE_2_ (Fig. 6B) or PGF_2α_ (Fig. 6C) treatment for 4 h (*P* < 0.05).

The PGE_2_ (Fig. 6B) -induced activation of CRE Luciferase was significantly reduced by treatment of FPS cells with the FP receptor (AL8810), EP2 receptor (AH6809), or EP4 receptor (ONOAE2227) antagonists or chemical inhibitors of PLC (U73122), PKA (4C3MQ), EGFR kinase (AG1478) or ERK1/2 kinase (PD98059), but not the PKC inhibitor (GF109203x; Fig. 6B *P* < 0.05). The PGF_2α_ (Fig. 6C) -induced activation of CRE Luciferase was significantly inhibited by co-treatment of cells with the FP receptor antagonist (AL8810) or chemical inhibitors of PLC (U73122), PKA (4C3MQ), EGFR kinase (AG1478) and ERK1/2 kinase (PD98059), but not the EP2 receptor antagonist (AH6809), EP4 receptor antagonist (ONOAE2227) or PKC inhibitor (GF109203x; Fig. 6C; *P* < 0.05).

## 4 Discussion

COX-2 expression is up-regulated in numerous pathologies including those of the reproductive tract such as ovarian carcinoma, cervical carcinoma and endometrial adenocarcinoma (Dore et al., 1998; Tong et al., 2000; Jabbour et al., 2001; Sales et al., 2001, 2002). The augmented biosynthesis of prostaglandins produced as a consequence of elevated COX-2 expression has been shown to promote tumorigenesis (Watanabe et al., 2000; Sonoshita et al., 2001; Seno et al., 2002) by interacting with specific prostaglandin receptors, which are also overexpressed in the same tumours.

We and others’ have shown that PGE_2_–EP receptor interaction can down regulate the expression of tumour suppressor genes (Sales et al., 2004c), increase cellular growth, migration and invasiveness (Sheng et al., 2001) and promote angiogenesis in in vitro and in vivo model systems (Watanabe et al., 2000; Sonoshita et al., 2001; Seno et al., 2002; Buchanan et al., 2003; Fujino et al., 2003; Sales et al., 2004a). Similarly PGF_2α_ can also enhance cell growth rate and induce the expression of inflammatory and angiogenic genes, in Ishikawa cells stably expressing the FP receptor and endometrial adenocarcinoma explants, via the FP receptor (Sales et al., 2004b, 2005; Jabbour et al., 2005).

We have recently reported elevated expression of FP receptor in endometrial adenocarcinomas (Sales et al., 2004b, 2005). Since endometrial pathologies biosynthesize PGE_2_ and PGF_2α_ in the micromolar range (Lundstrom and Green, 1978; Smith et al., 1981; Rees et al., 1984; Adelantado et al., 1988) which can act locally at the site of production on prostaglandin receptors and since PGE_2_ can bind to the FP receptor with an affinity that is only 10–30-fold less than PGF_2α_ (Abramovitz et al., 2000), we investigated the effect of PGE_2_ and PGF_2α_ on regulation of COX-2 via the FP receptor.

Using endometrial adenocarcinoma cells (Ishikawa cells) stably expressing the FP receptor to the levels observed in endometrial adenocarcinomas (FPS cells), we found that PGE_2_ dose-dependently mobilized InsP hydrolysis in FPS cells in a similar manner to that observed for PGF_2α_ via the FP receptor, since the specific FP receptor antagonist AL8810 abolished the PGE_2_ and PGF_2α_-mediated increase in InsP production. Although the PGE_2_-induced mobilization of InsP via the FP receptor was less than that observed for PGF_2α_, which we postulated was due to the reduced affinity of PGE_2_ for the FP receptor compared with the native ligand PGF_2α_, these data nevertheless demonstrate that elevated levels of PGF_2α_ as well PGE_2_ as can activate FP receptor signaling in tumors expressing elevated levels of FP receptor.

As FPS cells also express basal levels of EP2 and EP4 receptor (but not detectable EP1 or EP3 receptor), we found that PGE_2_ and to a much lesser extent PGF_2α_ could also mobilize intracellular cAMP. This effect of PGE_2_ on intracellular cAMP accumulation could be inhibited with the selective EP2 or EP4 receptor antagonists AH6809 and ONOAE2227 and abolished with the combination of EP2 and EP4 receptor antagonists. Interestingly only the EP2 receptor antagonist, but not the EP4 receptor antagonist abolished the PGF_2α_-mediated increase in cAMP. We found that neither the PGE_2_ nor PGF_2α_ effects on inositol phosphate hydrolysis and intracellular cAMP release was mediated by an intracellular mechanism involving activation of the downstream PKA, PKC or ERK1/2 pathways, as the chemical inhibitors of PKA, PKC and ERK1/2 failed to reduced the prostanoid mediated effects on InsP and cAMP accumulation.

The integrated response to GPCR coupling and second messenger activation results in phosphorylation of numerous effector signaling pathways, including the MAPK pathway (Naor et al., 2000), to regulate gene transcription. The MAPK pathway is a key signaling mechanism that regulates many cellular functions such as growth, differentiation and transformation (Lewis et al., 1998; Naor et al., 2000). We investigated the signaling pathways mediating the effect of PGE_2_ or PGF_2α_ on ERK1/2 in FPS cells. We found that PGE_2_ and PGF_2α_ stimulation of FPS cells induces ERK1/2 phosphorylation via identical intracellular signaling pathways via the PLC-PKA-mediated activation of the EGFR since co-treatment of cells with the PLC, PKA or EGFR kinase inhibitors significantly inhibited the PGE_2_ or PGF_2α_-induced ERK1/2 phosphorylation. We previously reported that PGF_2α_ signaling to ERK1/2 in FPS cells is mediated via the PKA and not PKC pathway (Sales et al., 2005). In the present study we demonstrate that the PGE_2_-mediated signaling to ERK1/2 in FPS cells is also PKA-dependent and PKC-independent, since the PKC inhibitor GF109203x failed to inhibit either the PGE_2_ or PGF_2α_-mediated signaling to ERK1/2. Moreover, we found that the PGE_2_-mediated effects on ERK1/2 are mediated largely in FPS cells via the FP receptor as the FP receptor antagonist inhibited the PGE_2_-mediated ERK1/2 phosphorylation to a greater extent than the EP2 or EP4 receptor antagonists. These data suggest that the cAMP pathway activated by the endogenous EP2/EP4 receptors acts synergistically with the InsP pathway to augment the signaling of PGE_2_ to ERK1/2 via the PKA-EGFR pathway.

We previously reported that PGF_2α_ could regulate COX-2 expression in an autocrine/paracrine manner to establish a positive feedback system for regulating endometrial tumorigenesis (Jabbour et al., 2005). In the present study we have shown that PGE_2_ can regulate COX-2 promoter activity and mRNA expression in a similar manner via the FP receptor as observed for PGF_2α_ via the PLC-PKA-EGFR-ERK1/2 signaling cascade. However, as observed for the PGE_2_-mediated effects on second messenger production and intracellular signaling reported herein, the PGE_2_-mediated increase in COX-2 expression via the FP receptor is also less than that produced by the native ligand PGF_2α_. We believe this difference is due to the lower binding affinity of PGE_2_ for the FP receptor compared with PGF_2α_ (Abramovitz et al., 2000). In addition, we found that the PGE_2_ activation of COX-2 promoter and mRNA expression was significantly reduced by the EP2 and EP4 receptor antagonists. This effect was not observed for PGF_2α_. Thus it would appear that unlike the PGF_2α_-mediated activation of COX-2 that occurs solely via the Gq activation of InsP, the PGE_2_ regulation of COX-2 in FPS cells is mediated by the synergistic effects of the cAMP and InsP second messenger systems via the PKA-EGFR-ERK1/2 pathway.

We next investigated the transcription regulatory regions within the COX-2 promoter activated by PGE_2_ and PGF_2α_ in FPS cells. The COX-2 promoter has binding sites for a number of transcription factors including nuclear factor (NF)-κβ, CCAAT/enhancer binding protein (C/EBP), AP-2 and cAMP response element (CRE) (Lukiw et al., 1998). Transfection studies with the *cis*-acting DNA binding sequence of the CRE or the full length C2.1 COX-2 promoter and a series of deletions containing key transcription factor-binding sites showed that promoter activity was maintained with a construct that had a CRE region only (RSA/HIN). Mutation of the CRE region of this construct resulted in complete loss of COX-2 promoter activity in response to administration of either PGE_2_ or PGF_2α_ indicating that the CRE is essential for transcriptional activation of the *COX-2* gene by prostaglandins. The regulation of the CRE by PGE_2_ and PGF_2α_ was further investigated by transient transfection studies using a cDNA construct containing the *cis*-acting DNA binding sequence of the CRE fused to a Luciferase reporter system. These studies showed that CRE activation in FPS cells is mediated by PGE_2_ and PGF_2α_ via the same mechanisms regulating COX-2, namely by activation of the PKA-EGFR-ERK1/2 pathways further confirming the importance of the CRE in the regulation of COX-2 activity in FPS cells.

Because PGE_2_ and PGF_2α_ mobilize intracellular cAMP and can activate protein kinase A (PKA) and since the PKA inhibitor 4C3MQ inhibited both the PGE_2_ and PGF_2α_-mediated signaling to ERK1/2 and COX-2 it is plausible that the mode of action of prostanoid signaling on activation of the CRE was by phosphorylation of CRE binding protein (CREB) at SER133, which can in turn bind the CRE and activate gene transcription. In the present study, we did not observe any significant phosphorylation of CREB at SER133 in response to agonist treatment by either PGE_2_ or PGF_2α_ by Western blot analysis (data not shown). Although activation of COX-2 by CREB binding to the CRE has been shown by Bradbury et al. (2003), Subbaramaiah et al. (2002a,b) have shown that COX-2 transcriptional activation via the CRE binding site can be mediated by activator protein (AP)-1. It is feasible that COX-2 transcriptional activation via the CRE in our study may be regulated via the binding of an alternative transcription factor or transcription factor complex to CREB, such as AP1.

In conclusion, our data provide strong evidence that the elevated biosynthesis of PGE_2_ and PGF_2α_ produced locally within endometrial adenocarcinomas can act in an autocrine/paracrine manner to enhance the expression of COX-2 via the CRE by means of their integrative actions on intracellular signaling pathways such as the ERK1/2 pathway. Moreover, we believe that these data have implications for the use of prostaglandin synthase inhibitors targeted against PGF synthase as therapeutic intervention strategies as suggested for PGE synthase (Murakami and Kudo, 2006; Jachak, 2007; Wang et al., 2006; Cheng et al., 2006). Our data highlight that in tumours expressing elevated levels of FP receptor, elevated biosynthesis of other prostanoids such as PGE_2_ can in the absence of the native ligand PGF_2α_ activate tumorigenic genes via the FP receptor. It is thus feasible that signaling pathways such as the EGFR or ERK pathways, which integrate the signaling from second messenger systems to target genes, may offer a better therapeutic target to reverse the adverse effects of prostanoid signaling, or indeed signaling in response to multiple prostanoids, in cancer.

## Figures and Tables

**Table 1:** List of reagents summarising the targets of each compound

Compound	Target	Reference
AL8810	FP receptor antagonist	Griffin et al. (1999)
AH6809	EP2 receptor antagonist	Woodward et al. (1995)
ONOAE2227	EP4 receptor antagonist	Mutoh et al. (2002)
U73122	PLC beta inhibitor	Bleasdale et al. (1990)
4C3MQ	Protein kinase A inhibitor	Lu et al. (1996)
GF109203X	Protein kinase C inhibitor	Toullec et al. (1991)
AG1478	Epidermal growth factor receptor tyrosine kinase inhibitor	Eguchi et al. (1998)
PD98059	Extracellular signal-regulated kinase kinase (MEK) inhibitor	Alessi et al. (1995)
